# YTH Domain Proteins: A Family of m^6^A Readers in Cancer Progression

**DOI:** 10.3389/fonc.2021.629560

**Published:** 2021-02-22

**Authors:** Yirong Xu, Wei Zhang, Feng Shen, Xi Yang, Huilan Liu, Shengbin Dai, Xinchen Sun, Junxing Huang, Qing Guo

**Affiliations:** ^1^ Department of Oncology, Taizhou People’s Hospital, Taizhou, China; ^2^ Graduate school, Dalian Medical University, Dalian, China; ^3^ Department of Neurosurgery, Taizhou People’s Hospital, Taizhou, China; ^4^ Department of Medical Oncology, Cancer Center, State Key Laboratory of Biotherapy, West China Hospital, Sichuan University, Chengdu, China; ^5^ Department of Radiation Oncology, The First Affiliated Hospital of Nanjing Medical University, Nanjing, China

**Keywords:** YTH domain, YTHDF1-3, YTHDC1-2, m^6^A modification, cancer

## Abstract

*N* ^6^-methyladenosine (m^6^A) is the most abundant internal modification in eukaryotic messenger RNAs (mRNAs). m^6^A RNA methylation is involved in all stages of RNA life cycle, from RNA processing, nuclear output, translation regulation to RNA degradation, indicating that m^6^A has various functions affecting RNA metabolism positively or negatively. Reading proteins are vital in regulating the translation and stability of m^6^A mRNAs positively or negatively. Recent studies have enhanced the understanding of the molecular mechanism of the YT521-B homology (YTH) domain family and the modification of m^6^A. This study aimed to review the specific mechanisms, functions, and interactions of the YTH domain protein family. It also discussed future research directions, thus providing new ideas for the clinical diagnosis and targeted therapy of cancer.

## Introduction

As early as in the 1970s, researches were conducted on *N* ^6^-methyladenosine (m^6^A) RNA (1). The m^6^A RNA modification is the most common internal modification of mammalian mRNAs, which occurs on the sixth N atom of base A (2). However, substantial evidence was lacking due to the immaturity of the technology and the instability of methylation modification at that time. Exploration of the genome-wide distribution of m^6^A remained unclear until 2011 when the first RNA demethylase named the fat mass- and obesity-associated protein (FTO) was identified (3), and the m^6^A RNA modification was found to be a dynamic and reversible process. Current knowledge of m^6^A has been established. The dynamic modification of m^6^A RNA methylation is completed by methyltransferase (“writers”) and demethyltransferase (“erasers”). Reading proteins (“readers”) recognize the m^6^A-modified mRNA and regulate the expression of downstream genes.

Surprisingly, an increasing number of studies have documented that m^6^A modification is crucial in regulating tumor function, and the dynamic imbalance of m^6^A modification can lead to the occurrence and development of tumor. Studies at the cellular level have shown that the dynamic modification of mRNAs is important in regulating mRNA splicing, output, stability, translation, and hence gene expression (4, 5). By extension, studies at the tissue and organ levels have demonstrated that m^6^A modification controls many biological processes, including spermatogenesis (6), neurogenesis (7), sex determination (8, 9), and stem cell self-renewal and life span (10, 11). Thus, the imbalance of the m^6^A pathway is often found in lung cancer (12), breast cancer (13), glioblastoma (14–16), acute myeloid leukemia (17), liver cancer (18), and other tumors. The regulatory mechanism of m^6^A and its biological functions have become a research hotspot in the field of RNA. But the influence of m^6^A on these biological processes and performing different functions in different settings still need further in-depth study.

**Figure 1:**
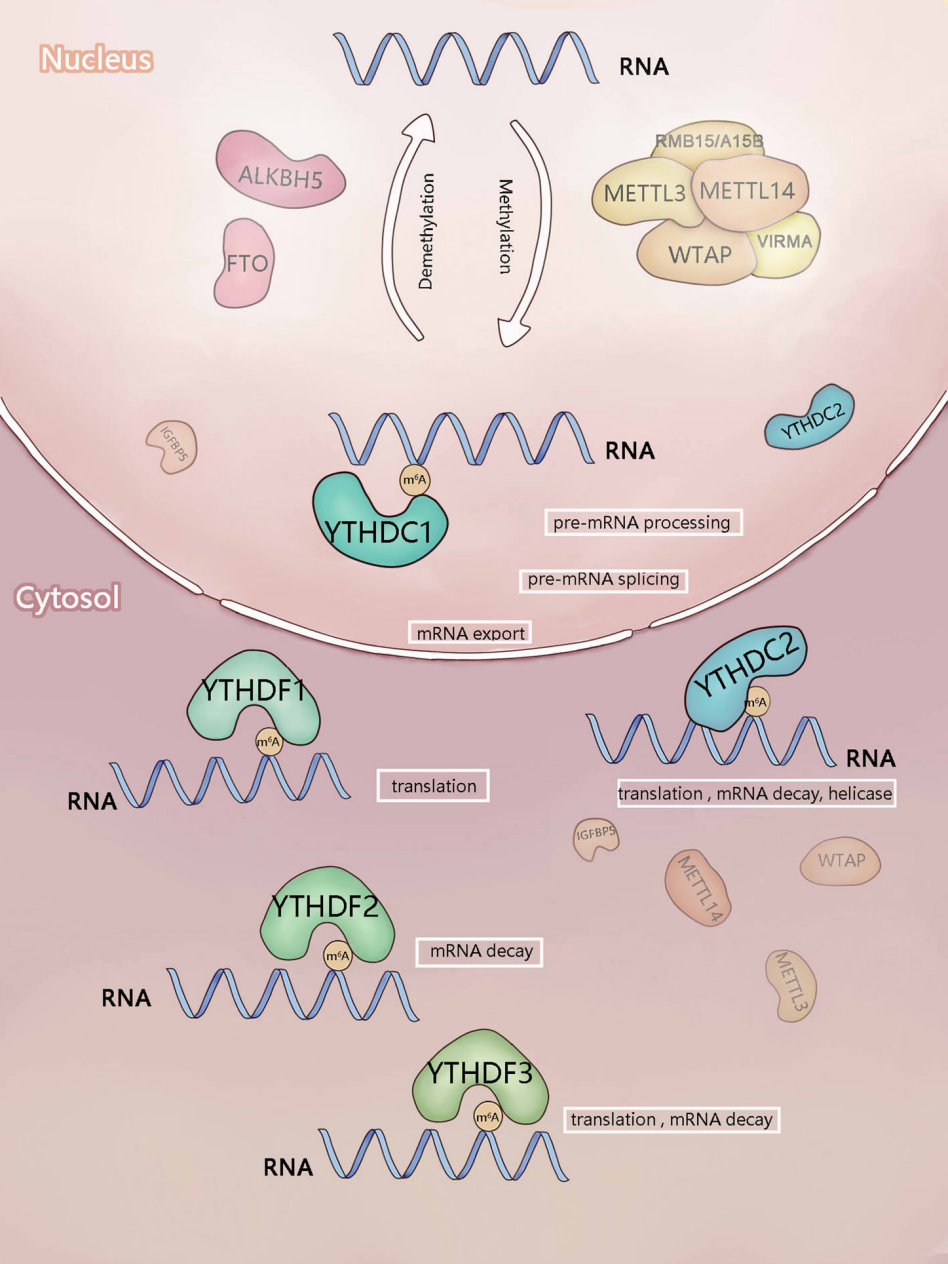
Components involved in m^6^A modification. m^6^A is mainly added by methyltransferase complex composed of methyltransferase-like3 (METTL3), METTL14),WTAP, RBM15/RBM15B, and VIRMA (writers). ALKBH5 and FTO (erasers) can remove m^6^A as demethyltransferase. In the nucleus, m^6^A can be recognized and regulated by YTHDC1 (reader) to regulate RNA splicing and mRNA export. In the cytosol, YTHDF1-3 and YTHDC2 (readers) recognize m^6^A, then they regulate the translation and stability of mRNA.

Methyltransferase and demethyltransferase make the mRNA dynamically and reversibly modified by m^6^A, while “readers” can recognize the methylated RNA and realize its function. Moreover, several different “readers” of m^6^A, including members of the YTH N6-Methyladenosine RNA Binding Proteins (YTHDFs) and YTH domain containing (YTHDCs) families, can recognize the modification of m^6^A and realize many biological functions specifically (2, 19–22) **(** **Table 1** and **Figure 1** **)**.

**Table 1:** Multiple functions exerted by YT521-B homology (YTH) family proteins in various tumor.

Cancer type	YTH domain proteins	Related target	Role in cancer	References
Liver cancer	YTHDF1		regulates the cell cycle and metabolism	(23)
	YTHDF2	SOCS2	promotes proliferation and migration	(24)
	YTHDF2	EGFR	inhibits liver cancer cell growth and proliferation	(25)
	YTHDF2	IL11 SERPINE2	Inhibits tumor-associated inflammation and tumor angiogenesis	(26)
Colorectal cancer	YTHDF1	Wnt/β-catenin pathway	promotes the occurrence and development of colorectal cancer	(27)
	YTHDF3	Gas5	promotes proliferation and metastasis	(28)
	YTHDC2	HIF-1α	promotes metastasis	(29)
Gastric cancer	YTHDF2		promotes cell proliferation and inhibit apoptosis	(30)
Esophageal cancer	YTHDC2	p53、NF-kappa B and JAK-STAT pathway	inhibits esophageal cancer cell growth	(31)
Ovarian cancer	YTHDF1	EIF3C	promotes tumorigenesis and metastasis	(32)
NSCLC	YTHDF1	CDK2 CDK4 cyclinD1	promotes NSCLC cell proliferation and xenograft tumor formation	(33)
	YTHDF2	6-PGD	enhance tumor growth	(34)
Pancreatic cancer	YTHDF2	YAP	inhibits migration and invasion	(35)
	YTHDF2	Akt/GSK3b/CyclinD1 pathway	promotes proliferation	(35)
Bladder cancer	YTHDF1 YTHDF3	ITGA6	promotes the growth and progression	(36)
	YTHDF2	SETD7 KLF4	promotes migration	(37)
AML	YTHDF2	TNFRSF2	promotes leukemic stem cells development and AML initiation	(38)
Breast cancer	YTHDF1 YTHDF3		poor prognosis	(39)
	YTHDF3	ST6GALNAC5、GJA1、EGFR VEGFA	Promotes brain metastasis	(40)
Melanoma	YTHDF1	HINT2	promotes cell growth and inhibits apoptosis	(41)

NSCLC, non-small cell lung cancer; AML, Acute Myeloid Leukemia.

The YTH domain family proteins comprise reading proteins widely distributed in the human body, and these “readers” are the major genes that mediate the function of m^6^A methylated RNAs. In this review, we summarize the up-to-date knowledge of ways the YTH domain family proteins affect biological functions in cancer and discuss their prospects as m^6^A regulators in cancer therapy.

## Mechanism of the YTH Domain Family Proteins

The YTH domain recognizes the m^6^A modification through a conserved aromatic ring. This is an RNA-binding domain dependent on m^6^A modification (42). Reading proteins recognize and read the information of m^6^A RNA in a methylation-dependent manner. YTHDC1-2 and YTHDF1-3 are the major Intracellular proteins in the human body. YTHDC2 is localized in the cytoplasm of meiotic spermatocytes, and YTHDF1-3 mainly recognizes the information of m^6^A methylation in the cytoplasm (21, 24, 44). These five proteins not only contain the same domain but also have their special domains that determine their different roles.

## YTHDF1

YTHDF1 promotes the translation of m^6^A RNA. Studies have shown that the main role of YTHDF1 is to accelerate the translation of m^6^A mRNA by promoting the ribosome assembly of m^6^A mRNA and interacting with the initiation factor, thereby regulating the translation kinetics (22) (**Figure 1**). For instance, YTHDF1 can increase the translation of Eukaryotic translation initiation factor 3 subunit C (ELF3C) and promote the overall translation output by combining with the m^6^A-modified ELF3C mRNA, thus promoting the occurrence and metastasis of ovarian cancer (32). In bladder cancer cells, YTHDF1 preferentially binds to the m^6^A mRNA of oncogene CUB domain containing protein 1 (CDCP1) to promote its translation, leading to the malignant transformation of urinary tract epithelium and occurrence of bladder cancer (45). YTHDF1 promotes the translation of Histidine triad nucleotide binding protein 2 (HINT2)-methylated mRNA but reduces the m6A modification in ocular melanoma (41). The extremely high or low level of m6A modification can lead to the occurrence of tumors.

YTHDF1 can selectively recognize the m^6^A RNA and bind to the GRAC (R is G or A) site, but not to other motifs near the m^6^A site (22). Snail family transcriptional repressor, the key transcription factor of EMT, has m^6^A methylation in its coding sequence (CDS) region. YTHDF1 is more likely to combine with the m^6^A RNA in CDS region to promote Snail translation (46). However, the binding sites of ELF3C mRNA and YTHDF1 are mainly located in coding region and untranslated regions in ovarian cancer cells (32). Furthermore, The YTHDF1-binding sites in dendritic cells are also enriched near the stop codon and 3’ untranslated regions (3’ UTRs) (47). A study on mouse hippocampus found that the binding sites of YTHDF1 were mainly located near the stop codons and 3’-UTRs (48). The methylation level of oncogene CDCP1 in the 3’ UTR significantly increased in bladder cancer cells (45).

Intriguingly, YTHDF1 is vital in promoting the translation initiation and translation elongation of m^6^A RNA. The binding of YTHDF1 to targeted RNA is selective. How this property is acquired and how YTHDF1 modifies downstream signaling pathways need further investigation.

## YTHDF2

Main function of YTHDF2 is to regulate the stability of m^6^A RNA. YTHDF2 mainly controls the life span and stability of target mRNA (**Figure 1**). The carboxyl-terminal domain of YTHDF2 is responsible for binding to the m^6^A RNA, and the amino-terminal domain is responsible for locating the YTHDF2 mRNA complex to the RNA decay site and transforming the m^6^A RNA from the translation state to the degradation state (21). Moreover, YTHDF2 destroys and further degrades the target mRNA by recruiting C-C motif chemokine receptor 4 (CCR4), not the deacetylase complex (49). It recognizes the m^6^A modification site in the 3’ UTR of Epidermal growth factor receptor (EGFR) mRNA, promotes its degradation (25), and plays an anti-tumor role. It inhibits the proliferation of hematopoietic stem cells by degrading the mRNA of self-renewal-related genes of hematopoietic stem cells (50). Furthermore, it targets Autophagy related 5 (ATG5) and Autophagy related 7 (ATG7) transcripts with a high level of m^6^A, resulting in mRNA degradation and thus reducing autophagy and adipogenesis (33). These studies suggest the involvement of YTHDF2 in biological processes by destroying the stability of key gene transcripts. In the heat shock stress response, YTHDF2 could promote the independent translation from the cytoplasmic localization to the protection of m^6^A at the 5’ UTRs end of the nucleus.

## YTHDF3

YTHDF3 can promote the translation and degradation of mRNA. YTHDF3 can promote the translation and degradation of m^6^A RNA (**Figure 1**). The function of YTHDF3 is both similar to and different from that of YTHDF2. YTHDF3 can locate the mRNA from the translation site to the explanation site by recognizing the m^6^A mRNA, and regulate its own mRNA translation to increase protein expression by itself (21, 51). Moreover, YTHDF3 can also interact with ribosomes and take part in the initial stage of translation (52). Further studies have shown that YTHDF3 induces the translation of m^6^A mRNA by binding with YTHDF1 and Eukaryotic translation initiation factor 4A3 (EIF4A3). The YTH domains at the C-terminus of YTHDF2 and YTHDF3 bind to the conserved G(m^6^A)C core motif, while the N-terminal domain is responsible for the migration of protein mRNA complexes to RNA decay sites (53).

## YTHDC1

YTHDC1 regulates the splicing of RNA. The YTH domain in YTHDC1 specifically recognizes the m^6^A modification and preferentially recognizes the G(m6A)C sequence (54). Then, YTHDC1 combines with the m^6^A-modified pre-RNAs to recruit the splicing factor Serine and arginine rich splicing factor 3 (SRSF3) but blocks the binding of Serine and arginine rich splicing factor 10 (SRSF10) to nuclear spots, thus promoting the splicing of exon inclusion bodies and mRNA output from the nucleus to the cytoplasm (20, 55) (**Figure 1**). YTHDC1 can recognize the m^6^A-modified chromosome-related regulatory RNAs (CarRNAs) [including promoter-related RNA (PaRNA), enhancer RNA (ERNA), and transposable element transcript RNA (repetitive RNA)], promote the decay and interpretation of CarRNAs, and regulate the state and transcription of chromatin.

## YTHDC2

YTHDC2 has many functions. YTHDC2 regulates the stability of m^6^A mRNA by recognizing the modification of m^6^A and recruiting the RNA degradation mechanism. It can also use its unique RNA-binding domain to build a bridge between m^6^A mRNA and ribosome and thus promote their effective translation. Among the YTH domain proteins, YTHDC2 is the only one containing the helicase domain; it is involved in meiosis (43, 56, 57). In addition, YTHDC2 is a protein with multiple domains. It may have complex functions in different cell environments, such as promoting translation, regulating the stability of transcripts, and serving as helicase (**Figure 1**). The interaction and regulation of these three functions remains unknown. Therefore, the contribution of these different functions in each field needs to be examined in more detail.

## Interactions in YTH Domain Family

In addition to their respective targeted mRNA sequences, YTHDF1 and YTHDF2 share a large group of targeted mRNA sequences, which can be regulated according to different cell signals. YTHDF1 can bind to the common sequence earlier (22). These results indicate that YTHDF1 promotes the translation of targeted mRNA, and YTHDF2 controls its life span. YTHDF1 and YTHDF2 regulate protein production and promote gene expression. The synergistic effect of YTHDF3 and YTHDF1 promotes protein synthesis and also affects the degradation of methylated mRNA mediated by YTHDF2. YTHDF3 may interact with the target mRNA before YTHDF1 and YTHDF2 (53). The three YTHDF proteins coordinate with each other to promote protein expression and have a certain degree of time sequence. The interaction between YTH domain family proteins can be regulated by m^6^A modification. Recent research confirms that m^6^A-modified mRNAs promote liquid-liquid phase separation (LLPS) of YTHDF proteins. On the contrary, mRNA-YTHDF complexes can regulate the translation efficiency and mRNA stability of m^6^A-modified mRNA (58).

The most widely studied and consensual mechanisms for YTHDF proteins were their ability to recognize m^6^A-mRNA, promote its degradation (YTHDF2/3) and translation (YTHDF1/3) specifically. A recent study has shown that no evidence demonstrates YTHDF proteins can promotes translation directly (59). In contrast, a finding suggests the opposite. m^6^A-Quantitative trait loci (QTL) mapping data analysis reveals that YTHDF proteins participate in different translational efficiency in a context-dependent manner. The investigators concluded that the translational efficiency depends on not only the sequence context surrounding the m^6^A site but also the binding site of other YTHDF proteins, rather than promoting translation simply (60). This is the first use of m^6^A-QTLs as a molecular genetic analysis tool for studies on m^6^A mechanisms. In our view, there are several potential explanations for this discrepancy. By using only YTHDF paralogs and different combinations of YTHDFs knockdown in HeLa cells, YTHDF proteins are believed to lead to mRNA degradation in the former study (59). This is inconsistent with our notion. Perhaps, additional experiments of this view are first required.

YTHDC1 is active in the nucleus, while YTHDC2 mainly functions in the cytoplasm. They are distributed in different functional areas and do not interfere with each other. Some functions of YTHDC2 are similar to that of the YTHDF proteins. However, a synergistic effect between them is still unclear.

## Techniques and Research Tools

YTH protein family regulates gene expression *via* selective combination of gene-specific m^6^A mRNA sequences and YTH proteins. Currently, there are several experimental approaches that can be used to elucidate the underlying mechanisms. To date, crosslinking and immunoprecipitation sequencing (CLIP-Seq) has been used to high-throughput approach of RNA binding protein interactions (61). However, the RNA-protein cross-linking efficiency of CLIP remains poor due to the low crosslink identification. Based on this, photoactivatable ribonucleoside-enhanced-crosslinking immunoprecipitation (PAR-CLIP) (62) and individual-nucleotide resolution CLIP (iCLIP) (63, 64) are optimized to give precise binding sites of YTH domains and transcripts (21, 22).

Moreover, Some databases help us to understand interactions within m^6^A reader proteins. RMBase, a new database, provides map of m^6^A modifications from epitranscriptome sequencing data (65, 66). Reliable m^6^A sites and transcriptional profiles were pooled by m^6^A-Atlas. m^6^A-Atlas database enables prediction of the biological function of every m^6^A site and inferences of pathogenesis of m^6^A-evoked disease (67). The M6A2Target database is a specialist database for estimating the prediction target of m^6^A modification (68). RNAWRE is the first database for finding effectors of RNA modifications (69). Knowing these database can help us gain a deeper insight of m^6^A modifications.

## Function of YTH Domain Family Proteins in Malignant Tumors

### Liver Cancer

The analysis of The Cancer Genome Atlas (TCGA), Gene Expression Omnibus (GEO) databases and the immunohistochemical analysis have shown that YTHDF1 is upregulated, associated with poor overall survival (OS) and pathologic staging of liver cancer. YTHDF1 is an independent adverse prognostic factor involved in regulating the cell cycle and metabolism of liver cancer (23, 70, 71). Therefore, it is considered to be a cancer-promoting factor. The expression of YTHDF2 in Hepatocellular Carcinoma (HCC) increases, which is closely related to the malignant degree of HCC. Research shows that miR-145 can regulate the expression of YTHDF2 and thus inhibit the proliferation of HCC cells (72). Another study showed that m^6^A modifications of Cytokine Signaling 2 (SOCS2) mRNA can be facilitated by methyltransferase-like3 (METTL3), and the degradation process of SOCS2 can be accelerated by YTHDF2 during HCC progression (24). Other studies have shown that YTHDF2 can be regarded as a tumor suppressor, inhibiting the growth of hepatoma cells. YTHDF2 promotes mRNA degradation of EGFR by binding to m6A site within the EGFR 3’UTR, simultaneously inhibiting proliferation of HCC cells. YTHDF2 inhibits inflammasome-mediated tumor vascularization and malignancy by degradation of m^6^A-containing serpin family E member 2 (SERPINE2) and interleukin-11 (IL-11) mRNA (25, 26). Also, YTHDF2 has a contradictory role in different research backgrounds. Future studies should demonstrate the specific effect of YTHDF2 on liver cancer and the involved signaling pathway, and also determine whether YTHDF2 is a tumor suppressor or a cancer-promoting factor. The participation of other members of the YTH family, such as YTHDC1 and YTHDC2, in the development of liver cancer has not yet been explored.

### Colorectal Cancer

Through the analysis of cancer tissues and *in vitro* experiments, some researchers found that YTHDF1 was related to the behavior of a variety of malignant tumors and affected the sensitivity of colorectal cancer to chemotherapeutic medicines; also, the carcinogenic factor c-myc could regulate the expression of YTHDF1 (73). The results of Bai et al. also suggested that the high expression of YTHDF1 in colorectal cancer promoted the occurrence and development of colorectal cancer through Wnt/β-catenin pathway (27). Therefore, YTHDF1 can be used as a cancer-promoting factor. Low expression of YTHDF1 was found to be correlated with the infiltration of CD8+ T cells in colorectal carcinoma (CRC) tissue (47). The data suggest that YTHDF1 may have contributed to the regulation of tumor immune microenvironment. Another study demonstrates that lncRNA Gas5 inhibits cellular proliferation and metastasis of CRC by promoting Yes-associated protein (YAP) phosphorylation and YAP-mediated YTHDF3 transcription. Moreover, YTHDF3 binds to the m6A-modified lncRNA Gas5 and inhibits lncRNA Gas5 decay, thus generating a negative feedback loop (28). The high expression of YTHDC2 is related to the malignancy of colon cancer and contributes to tumor metastasis (29). However, YTHDC2 does not play the role of a reading protein and has nothing to do with the modification of m^6^A. YTHDC2 promotes the translation of hypoxia-inducible factor-1 α (HIF-1α) and the metastasis of colorectal cancer cells by playing the role of helicase.

### Esophageal and Gastric Cancer

*In vitro* studies have shown that the knockdown of YTHDF2 can inhibit the proliferation of gastric cancer cells and promote their apoptosis (30). YTHDF2 can be used as a new target to study the mechanism of gastric cancer. As a suppressor of esophageal cancer, YTHDC2 is lowly expressed in esophageal cancer. Furthermore, high expression of YTHDC2 can inhibit the growth of tumor cells *in vitro.* A single nucleotide polymorphism named Rs2416282 is localized at YTHDC2 promoter and regulates YTHDC2 expression. In addition, Kyoto Encyclopedia of Genes and Genomes (KEGG) enrichment analysis about the regulation of esophageal squamous cell carcinoma (ESCC) by YTHDC2 revealed that YTHDC2 is associated with p53, NF-kappa B and JAK-STAT signaling pathways (31).

### Ovarian Cancer

The public database of ovarian cancer and *in vitro* and *in vivo* experiments showed that the expression of YTHDF1 was related to the stage and prognosis of ovarian cancer. Eukaryotic Translation Initiation Factor 3 Subunit C (EIF3C) exerts its oncogenic function by promoting its translation in an m6A-dependent manner that lead to tumor growth and metastasis in ovarian cancer (32). The occurrence of ovarian cancer is related to the modification of m^6^A. YTHDF1 can be used as a new target of ovarian cancer-targeted diagnosis and treatment. The effect of other m6A-modified related proteins on ovarian cancer can be explored in future studies.

### Lung Cancer

Studies on nonsmall cell lung cancer (NSCLC) have shown that the high expression of YTHDF1 can promote the development of tumors by promoting protein translation of cyclin E-associated kinase 2 (CDK2), cyclin-dependent kinase 4 (CDK4) and cyclin D1 mRNAs. Furthermore, low expression of YTHDF1 can reduce the sensitivity of cancer tissue to cisplatin was attributed to the Keap1-Nrf2-AKR1C1 axis (Kelch-like ECH-associated protein 1, NF-E2 p45-related factor 2 and antioxidant genes including aldo-keto reductases 1C1) (33). This study suggested that YTHDF1 was related to lung cancer.

The role of YTHDF1 in cancer occurrence and treatment remains controversial. YTHDF1 is involved in biological processes *via* different molecular mechanisms. It is related to the sensitivity to cisplatin. However, whether ITF1 has the same effect as other chemotherapy medicines is still unclear. All these problems need exploration. YTHDF2 binds to the m^6^A modification site of 6-phosphogluconate dehydrogenase (6PGD) 3’-UTR and facilitate 6PGD mRNA translation in lung cancer cells. Based on this, the authors suggest that YTHDF2 can promote the growth of lung cancer by affecting the pentose phosphate pathway (PPP) (34). Recent studies have shown that YTHDF1 and YTHDF2 are highly expressed in lung adenocarcinoma and related to the prognosis of patients, but the specific mechanism remains unclear (74). Data from The Cancer Genome Atlas (TCGA) and Genotype-Tissue Expression (GTEx) database show that both YTHDF1 and YTHDF2 were highly expressed in lung adenocarcinoma (75). Alkylation Repair Homolog 5 (ALKBH5) inhibits tumor growth and metastasis by reducing m^6^A modification of YAP and suppressing activity of miR-107/Large Tumor Suppressor Kinase 2 (miR-107/LATS2)-mediated YAP. YTHDF proteins may play a critical role in this process (76).

### Pancreatic Cancer

A study on pancreatic cancer demonstrated the high expression of YTHDF2. YTHDF2 inhibited adhesion, invasion, migration, and Epithelial-Mesenchymal Transition (EMT) through the YAP signal and promoted the proliferation of pancreatic cancer cells through the Serine/threonine-protein kinases/Glycogen synthase kinase 3 beta/Cyclin D1 (AKT/GSK3B/CCND1) pathway (35). YTHDF2 has dual effects on pancreatic cancer. YTHDF2 can be used as a new marker of pancreatic cancer, but the specific mechanism needs to be clarified. YTHDC2 is also considered to be a frequently mutated gene in patients with pancreatic cancer. We conclude that YTHDC2 may be associated with pancreatic cancer susceptibility (77).

### Bladder Cancer

The combination of YTHDF1 and YTHDF3 promotes the translation of Integrin subunit alpha 6 (ITGA6) mRNA and the development of bladder cancer (36). YTHDF2 promotes the mRNA degradation of tumor suppressor genes SET domain containing lysine methyltransferase 7 (*SETD7)* and Kruppel Like Factor 4 (KLF4) by identifying METTL3-mediated m^6^A modification, thus inducing bladder cancer progression (37). METTL3/YTHDF2-SETD7/KLF4 m^6^A axis plays an important role in bladder carcinogenesis. Many reading proteins of the YTH family are related to bladder cancer; they regulate the tumor tissue by influencing different target genes downstream positively or negatively.

### Leukemia

YTHDF2 is involved in regulating the self-renewal of hematopoietic stem cells (HSCs) (50, 78). The abnormal expression of YTHDF2 may relate to some hematologic neoplasms. One study has demonstrated that inhibited YTHDF2 selectively compromises acute myeloid leukemia (AML) (38). YTHDF2 can be used as a potential target in the treatment of leukemia.

### Breast Cancer

It has been shown that higher YTHDF1 and YTHDF3 expressions were correlated with a shorter survival in breast cancer (BC) (39), and the importance of YTHDF3 as an independent risk factor for BC has been a topic of significant research. Another study has proposed that overexpressed YTHDF1 and YTHDF3 promotes translation of oncogenes in a m6A-dependent manner in BC, resulting in tumor progression and inferior outcomes (51). YTHDF3 expression in BC brain metastases is higher than that in BC and nontumor tissues, and facilitates expressions of ST6 N-Acetylgalactosaminide Alpha-2,6-Sialyltransferase 5 (ST6GALNAC5), gap junction proteinα1 (GJA1), EGFR, and vascular endothelial growth factor A (VEGFA) in a m^6^A-dependent manner during the regulation of multiple steps in brain metastases (40).

### Melanoma

Initial study on melanoma demonstrated that YTHDF1 can promote translation by binding Histidine Triad Nucleotide Binding Protein 2 (HINT2) transcripts, that in turn promotes tumor cell proliferation, inhibits apoptosis and confers a poor prognosis (41). Of interest, the increased m^6^A methylation of important oncogenes in FTO knockdown cells also promoted YTHDF2-induced degradation in melanoma, whereas melanoma cells appear to be more sensitive to IFN-γ and enhance the efficacy of melanoma immunotherapy (79).

**Figure 2:**
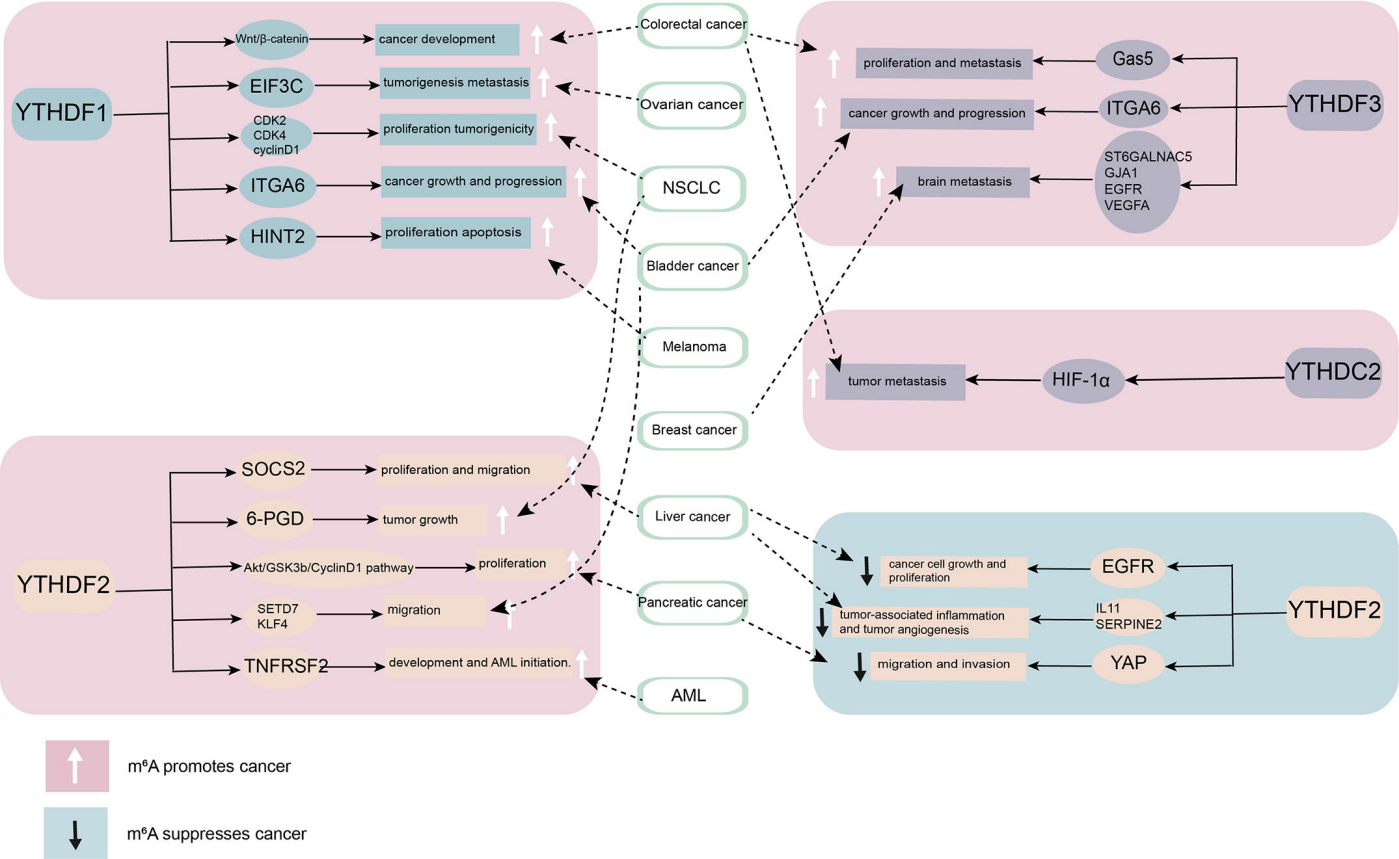
The role of YT521-B homology (YTH) domain family proteins in cancer. By combining different downstream targets, YTH domain family proteins promote or inhibit the occurrence and development of cancer. YTHDF1, YTHDF3, and YTHDC2 mainly play a role as cancer promoting factor. YTHDF2 plays diverse roles in different cancers.

The abnormal expression of YTH family proteins in various tumor tissues is related to the occurrence of tumors and promotes the growth and metastasis of tumors **(** **Figure 2** **)**. The role of the YTH domain protein family in tumor target tissue is preliminarily understood; it can be used as a potential for later research. However, different mechanisms exist in different research backgrounds. Hence, extensive investigation is needed to clarify the specific mechanism underlying the involvement of YTH family proteins in tumors as the target of treatment. The available findings suggested that the mRNA and protein levels of YTH family proteins could be simultaneously examined. The questions to be addressed were as follows: Whether the involvement of reading proteins varies in different tumors at the RNA level due to different splicing and maturation processes controlled by upstream signals; whether the binding to the m^6^A mRNA is related to the surrounding environment at the protein level, leading to the different roles of m^6^A in different stages of tumor development; The relationship between YTH family proteins and cancer is still unknown to a large extent. These findings provide novel ideas for the study of epigenetics of cancer and hence may help find new targets for antitumor therapy.

**Figure 3:**
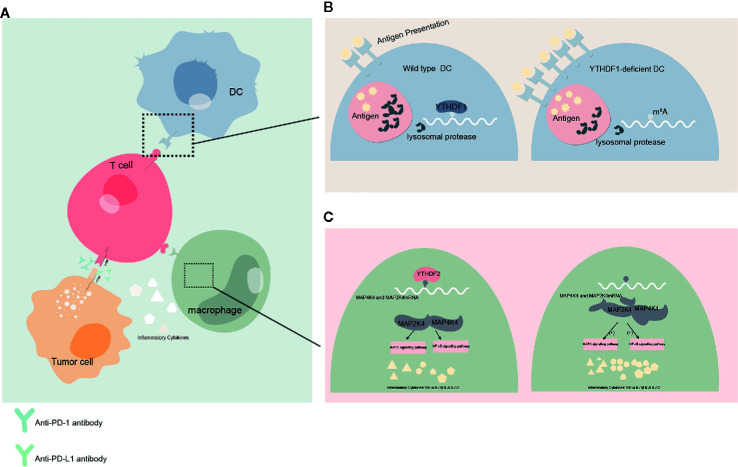
Effect of YTHDFs on tumor immune microenvironment (TIME) **(A)** Dendritic cells (DC)**** presents the antigen to the surface of T cells and initiates the adaptive immune response. Programmed cell death protein 1 (PD-1)/programmed death-ligand 1 (PD-L1) is a negative immune checkpoint pathway that inhibit immune responses and promotes immune escape of tumor cells. This process can be blocked by anti-PD-1/PD-L1 antibodies. Macrophages can affect anti-tumor immunity by secreting inflammatory factors. **(B)** When YTHDF1 is deficient, the translation efficiency of lysosomal protease transcripts decreases, the degradation of antigens is reduced, more antigens are involved in antigen presentation, and the immune response of T cells is enhanced. This process may affect the efficacy of PD-L1 checkpoint inhibitors. **(C)** In macrophages, YTHDF2 knockdown promotes the translation and stability of MAP2K4 and MAP4K4 mRNA, which activates MAPK and NF-κB signaling pathways and promotes the expression of pro-inflammatory cytokines. These inflammatory factors can participate in the regulation of immune response and affect the efficacy of anti-tumor immunity.

These studies show not only that YTH protein family has significant effects on regulating the development and function of tumor, but also that YTH protein family can influence the antineoplastic treatment. How YTH protein family might impact cancer immunotherapy will be important to examine in the future. In the current study, YTHDF1 have the potential to identify and translate m6A-modified lysosomal protease, which suppress cross-presentation activity in dendritic cells (DC) and weakens the sensitization to CD8+ T cells. This could suggest a potential underlying mechanism of immune escape (47). Taken together, the current study suggests that YTHDF1 may be harnessed as a new immunotherapeutic target to improve patient outcomes with cancer. In another study conducted in head and neck squamous cell carcinoma implicates that YTH domain protein family are related to tumor immune microenvironment (TIME), whereas PD-L1 upregulation is associated with m^6^A methylation and patients with high-risk scores may receive more benefit from radiotherapy (80). Meanwhile YTHDF2 also appear to exert negative regulation in macrophages-induced inflammatory responses (81) (**Figure 3**).

Oxygen content in tumor cells effects radiosensitivity directly. It has been demonstrated that YTHDF1 is associated with hypoxia adaptation (33). Moreover, m6A-modified METTL3 is involved in the influence of DNA damage response upon UV radiation (82). These results suggest that m^6^A modifications might also influence the radiosensitivity through DNA damage and/or DNA repair. Therefore, the mechanisms of YTH family proteins effect on radiosensitivity are ongoing in our laboratory

## Future Prospects

RNA m^6^A modification is a dynamic and reversible modification catalyzed by methyltransferase, demethylase, and reading proteins. Readers can link RNA modification at specific sites with specific regulatory functions in cells. Although the recent progress of YTH family proteins in cancer has been summarized previously in review form (83), we conclude by summarizing the most important findings about mechanisms, functions, and interactions of YTH protein family from a different perspective.

The research on the role of YTH domain family proteins in cancer has greatly advanced, but is still associated with various challenges. First, the mechanism of YTH domain family proteins in cancer research is still unclear to a large extent. Second, whether YTH domain family proteins can be used as potential targets for cancer diagnosis and treatment, as well as their specificity and sensitivity, needs to be further explored. Also, some studies have shown that the YTH family proteins and their related new signaling pathways can be used. However, the lack of large-sample clinical studies and the side effects of targeted therapy are unknown.

With recent striking combination treatment effects of cancer-immunotherapy (40, 47), identifying m^6^A-related immunotherapy-sensitive tumor types and understanding how m^6^A modifications influence immunotherapy sensitivity and radiosensitivity are urgently needed.

The continuous progress of high-throughput sequencing technology, RNA-Seq technology, and immunoprecipitation technology has provided a deeper understanding of the m6A modification pathway and indicated an important biological function of m6A by regulating RNA stabilization, localization, transportation, splicing, and translation. The research on the involvement of YTH domain family proteins in cancer and other diseases has provided new ideas and laid a theoretical foundation for developing a therapeutic strategy for the use of YTH domain family proteins in cancer.

## Author Contributions

YX and WZ contributed to the drafting and editing of the manuscript, and shared the first authorship. QG and JH designed, revised, and finalized the manuscript. HL, FS, XY, SD, XS, LO, and DC participated in the drafting and editing of the manuscript. XS assisted in the revision of the manuscript. YX contributed to literature search. All authors contributed to the article and approved the submitted version.

## Funding

This work was supported by the Innovative Team of Jiangsu Province (CXTDA2017042), China Postdoctoral Science Foundation (2019M663505), and Taizhou science and technology support plan (Social Development) project (TS201903).

## Conflict of Interest

The authors declare that the research was conducted in the absence of any commercial or financial relationships that could be construed as a potential conflict of interest.
